# Increased Risk of Transition to Institutional Care Among Community‐Dwelling Older Adults With Cognitive Frailty: A Competing Risks Survival Analysis

**DOI:** 10.1002/gps.70197

**Published:** 2026-02-03

**Authors:** Jinwei Bian, Zi Chen, Daniel Yee Tak Fong, Edmond Pui Hang Choi, Pui Hing Chau

**Affiliations:** ^1^ School of Nursing The University of Hong Kong Hong Kong China

**Keywords:** cognitive frailty, cognitive impairment, institutional care, nursing home admission, physical frailty

## Abstract

**Background:**

Cognitive frailty (CF) is the coexistence of physical frailty and cognitive impairment. Transition to institutional care (TIC) refers to the move from home to a long‐term care institution and represents a major change in living arrangement and care needs among older adults. Both CF and TIC are pressing challenges in ageing populations; however, evidence on their association remains limited.

**Objectives:**

This study aimed to explore the longitudinal relationship between CF and TIC among community‐dwelling older adults, using the Chinese population as an example.

**Methods:**

This retrospective cohort study utilised data from four waves (2008–2018) of the Chinese Longitudinal Healthy Longevity Survey. Community‐dwelling participants aged between 65 and 100 years at baseline were included. CF was defined based on the modified Fried criteria and the Chinese version Mini‐Mental State Examination. The Fine‐Grey subdistribution regression models were used, treating mortality and lost to follow‐up as competing risks and controlling for gender, age, living area, marital status, living arrangement, multimorbidity, household income, and preference for institutional care.

**Results:**

The baseline prevalence of CF was 2.3% (95% CI: 2.0%–2.6%). During follow‐up, 1.2% (95% CI: 1.0%–1.4%) transitioned to institutional care, 47.1% (95% CI: 46.1%–48.2%) died before TIC and 32.1% (95% CI: 31.1%–33.0%) were lost to follow‐up. Incidence rate of TIC was 2.3 (95% CI: 1.9–2.8) per 1000 person‐years. Individuals with CF had a higher risk of TIC (SHR 3.51, 95% CI: 1.49 to 8.28; *p* = 0.004) compared to those without physical frailty and cognitive impairment.

**Conclusion:**

Our findings demonstrated the positive association between CF and TIC, highlighting the need for appropriate and timely management of CF and personalised interventions for this vulnerable group to delay premature institutionalisation.

## Summary

- The prevalence of cognitive frailty was 2.3% among community‐dwelling older adults without dementia.
- The incidence of transition to institutional care was 2.3 per 1000 person‐years.
- Cognitive frailty was associated with an increased risk of transition to institutional care, as well as higher risks of mortality and lost to follow‐up compared to individuals without physical frailty and cognitive impairment.
- Preventive interventions are required to reduce the risk of developing cognitive frailty and the occurrence of cognitive frailty related adverse outcomes.

## 1 Introduction

Population ageing is a global public health issue, with far‐reaching implications for both clinical practice and long‐term care. According to the World Population Prospects 2023, approximately 9.6% of the world's population was over 65 years old in 2021, and this proportion is projected to rise to 16.5% by 2050 [1]. In China, the proportion of people aged over 60 is projected to reach 28% by 2040 [2]. While the rapid population ageing, giving an increasing size of the older population, may increase the demand for institutional care [3], this trend may be partly offset by healthy ageing and ageing‐in‐place [4, 5].

Transitional care, defined as the movement between healthcare settings or levels of care, commonly involves older adults relocating from home to an institution—a process known as transition to institutional care (TIC) [6]. Prior studies have linked TIC to adverse outcomes such as hospitalisation and mortality [7, 8]. Two Chinese studies reported that older adults transitioning to institutional care often exhibited greater health burdens, such as cognitive and functional decline, higher dependency, and multiple chronic conditions [9, 10]. Additionally, many older adults prefer to age in place [11], as they have greater autonomy in a familiar environment [12, 13].

Cognitive frailty (CF), proposed by the International Academy of Nutrition and Ageing (IANA) and the International Association of Gerontology and Geriatrics (IAGG) in 2013, refers to the simultaneous presence of physical frailty and cognitive impairment (clinical dementia rating [CDR] = 0.5), excluding concurrent Alzheimer's disease or other dementias [14]. According to a systematic review of 24 studies, the prevalence of CF ranged from 2.4% to 50.1% in Asia, 5.4%–12.1% in North America, and 1.0%–22.6% in Europe, with regional variations possibly due to differences in sample characteristics and investigation times [15]. One meta‐analysis of 15 studies indicated that CF was an independent risk factor for adverse outcomes, including falls, disability, and hospitalisation [16]. Another systematic review of 14 studies reported that older adults with CF were at increased risk of mortality, dementia, decreased activity and poor quality of life [17].

A scoping review of 34 studies summarised health‐related factors—such as cognitive dysfunction and frailty—as one category of predictors associated with nursing home admission, with most included studies conducted in the United States and Europe [18]. Another scoping review of 204 studies—103 from the United States and only 5 from China—similarly identified cognitive impairment and frailty as key predictors of TIC [19]. These findings suggest that CF may contribute to early TIC. While CF may offer a distinct perspective compared to studies focussing on either factor alone, no studies have specifically examined the relationship between CF and TIC. Two prior studies in Japan and South Korea showed that CF was a significant predictor of long‐term care needs among community‐dwelling older adults [20, 21]; however, they did not distinguish between institutional and community‐based care, nor did they assess actual utilisation. Hence, there was a research gap in understanding the relationship between CF and institutionalisation, which our study aimed to address.

This research direction is particularly important because individuals with CF, who experience both physical and cognitive decline but do not meet the diagnostic criteria for dementia, represent a clinically distinct and potentially modifiable group [22]. Exploring the association between CF and TIC may provide valuable insights into care trajectories before the onset of dementia and help identify opportunities for early intervention to delay or prevent institutionalisation. Moreover, excluding individuals with dementia is essential when exploring this relationship, as the definition of CF inherently excludes those with established dementia [14].

Therefore, this study aimed to explore the longitudinal relationship between CF and TIC among community‐dwelling older adults without dementia, using the Chinese population as an example. Our study is based on a conceptual framework that both frailty and cognitive impairment contribute to TIC [19, 23]. Physical frailty increases dependence by limiting mobility and functional capacity, while cognitive impairment hinders the ability to manage daily tasks independently [19, 23]. The combination of these two conditions may overwhelm informal caregivers, particularly in settings where professional home care is limited or unavailable, thereby substantially increasing the risk of TIC. In China, demographic shifts and weakening family support systems may further elevate the risk of TIC among older adults with CF [24]. By understanding the association between CF and TIC, individualised interventions for older adults with CF may be developed to support ageing in place and thereby improve health‐related quality of life.

## 2 Materials and Methods

### 2.1 Data and Sample

The data used in this study were derived from the Chinese Longitudinal Healthy Longevity Survey (CLHLS), a national survey of older adults aged 65 and above [25]. A multi‐stage cluster random sampling method was adopted to recruit participants from 23 out of 34 provinces in China, providing representative data to investigate determinants of longevity. The baseline survey was conducted in 1998, and the follow‐up surveys were conducted in 2000, 2002, 2005, 2008–2009, 2011–2012, 2014 and 2017–2018. CLHLS was approved by the Research Ethics Committee of Peking University (IRB00001052–13074).

The study sample comprised older adults who were interviewed in 2008 (wave 1). Inclusion criteria for this current analysis were: (1) between 65 and 100 years at baseline, (2) followed up in at least one of the three waves: 2011/2012 (wave 2); 2014 (wave 3) and 2017/2018 (wave 4). Exclusion criteria were: (1) living in any form of institutional care at baseline, (2) unable to determine the status of CF at baseline, (3) missing data on covariates at baseline, (4) self‐reported or cognitive test‐based dementia at baseline [26]. Self‐reported dementia was defined as an affirmative response to the question, ‘do you suffer from dementia?’. Cognitive test‐based dementia was defined by a score of the Chinese version of Mini‐Mental State Examination (MMSE‐C) below the age‐ and education‐specific cutoff (Supporting Information S1: Table S1). Participants who met either criterion were considered to have dementia.

### 2.2 Measurements

#### 2.2.1 Transition to Institutional Care (TIC)

TIC was measured by the question on co‐residence status. People who answered, ‘in an institution (including nursing home, residential care home, old age home, etc.)’ in any of the follow‐up waves were considered as TIC. Time‐to‐event was defined as the time interval from baseline to the time that TIC occurred. As the actual time of TIC was not available, the time was assumed to be the midpoint between the last wave without residence in institution and the subsequent wave with institutional residence [27]. For example, if no event was reported in 2011 but was first observed in 2014, the proxy time‐to‐event was 4.5 years from baseline (2008). This proxy was used instead of using interval‐censored time‐to‐event because the chosen statistical model could not accommodate two competing events.

#### 2.2.2 Competing Risks

Mortality and lost to follow‐up were taken as two competing risks, which were ascertained at each follow‐up wave. As three mutually exclusive events (TIC, mortality and lost to follow‐up), the occurrence of one event is often precluded by other two events. Mortality terminates the observation period, whereas lost to follow‐up leads to premature truncation, both affecting the assessment of TIC. To account for these competing events and reduce potential bias, we applied a competing risk approach [28]. Information on each event was collected in 2011/2012 (wave 2), 2014 (wave 3) and 2017/2018 (wave 4). For individuals who did not experience any of the events of interest during the study period, observations were censored at their last follow‐up in wave 4.

#### 2.2.3 Frailty and Cognitive Impairment Status

Older adults with both physical frailty and cognitive impairment in the absence of dementia were considered CF [14]. Physical frailty was defined as participants who met ≥ 3 of the modified Fried criteria which consisted of five domains: exhaustion, shrink, weakness, low mobility, and inactivity [29, 30]. Cognitive impairment and dementia were identified if participants had a MMSE‐C score below the corresponding age‐ and education‐specific cut‐off (Supporting Information S1: Table S1) [31]. Participants who answered ‘not able to answer’ were treated as missing. The MMSE‐C score cutoff for mild cognitive impairment aligned with the definition of CDR = 0.5 [32].

Despite the presence of missing items, physical frailty and cognitive impairment were not treated as missing when their status could be identified based on non‐missing items. Based on the above assessment, participants were classified into four groups: (1) unimpaired (participants without physical frailty nor cognitive impairment); (2) physical frailty only (participants with physical frailty only but not cognitive impairment); (3) cognitive impairment only (participants with cognitive impairment only but not physical frailty); (4) CF (participants with both physical frailty and cognitive impairment).

#### 2.2.4 Covariates

Based on previous literature [18], data availability, and modelling requirements, we selected a set of covariates, including gender, age group, living area, education level, marital status, living arrangement, multimorbidity, household income and preference for institutional care.

### 2.3 Statistical Analysis

Descriptive statistics for participants' baseline characteristics were presented, and incidence rates of three events per 1000 person‐years were calculated. The chi‐square test and one‐way analysis of variance (ANOVA) were used to investigate differences in baseline characteristics and follow‐up status across different CF status groups.

To examine the association between CF and TIC, we used the subdistribution hazards model to estimate subdistribution hazard ratios (SHRs) and 95% confidence intervals (CIs) [28]. In this study, SHR greater than 1 indicates an increased risk of TIC compared to the reference group, while values less than 1 indicate a reduced risk. The subdistribution hazards model was proposed by Fine and Grey, in which the hazard function for a given event is the instantaneous rate of occurrence of that event in subjects who are either currently event‐free or who have already experienced a concurrent event [28, 33]. We chose this model because it accounts for both competing risks and censoring, thereby providing valid estimates of the cumulative incidence of the event of interest (TIC). We first fitted a subdistribution hazards model with TIC as the event (Model 1), treating death and lost to follow‐up as competing events and adjusting for covariates described above. To further examine the excess risk associated with CF, we performed two pairwise comparisons: CF versus physical frailty only, and CF versus cognitive impairment only. Details of the assessment of intra‐class correlations across provinces are provided in Supporting Information S1: Text S1. To understand the care trajectories and justify the inclusion of competing events in the main analysis, subdistribution hazards models for mortality (Model 2) and lost to follow‐up (Model 3) were also developed, treating the other two events as competing risks and adjusting for the same set of covariates.

We performed five sensitivity analyses to examine the robustness of the main results. First, CF was redefined using the Frailty Index (FI) and MMSE‐C. Second, items with ‘not able to answer’ responses in the MMSE‐C were reconsidered as incorrect answers. Third, time‐to‐event was redefined as the interval from the baseline survey to the date of the follow‐up survey at which the first event was observed. Fourth, participants lost to follow‐up were treated as censored observations. Fifth, missing data on CF status and covariates were handled using multiple imputation.

The proportional hazards assumption was satisfied for all variables, and no evidence of multicollinearity was detected (variance inflation factor < 2) [34]. Analysis was performed using SPSS version 27, and a two‐sided level of significance of 0.05 was used.

## 3 Results

### 3.1 Cohort Characteristics

Of the 15,925 participants, 8715 were included in the study sample (Supporting Information S1: Figure S1). The prevalence of CF was 2.3% (95% CI: 2.0%–2.6%), and age, gender, marital status and educational level were significantly associated with CF groups (*p* < 0.05) (Table 1).

**TABLE 1:** Baseline characteristics of participants in the sample.

Variables	All	Unimpaired	Physical frailty only	Cognitive impairment only	CF	*p*‐value
No. of participants	8715	6668 (76.5%)	1109 (12.7%)	740 (8.5%)	198 (2.3%)	—
Age (mean ± SD)	82.1 ± 9.5	80.8 ± 9.3	89.0 ± 7.2	80.5 ± 10.0	89.8 ± 7.4	< 0.001
65–74	2326 (26.7%)	1984 (29.8%)	48 (4.3%)	282 (38.1%)	12 (6.1%)	
75–84	2590 (29.7%)	2172 (32.6%)	218 (19.7%)	173 (23.4%)	27 (13.6%)	
≥ 85	3799 (43.6%)	2512 (37.7%)	843 (76.0%)	285 (38.5%)	159 (80.3%)	
Gender						< 0.001
Female	4140 (47.5%)	3006 (45.1%)	640 (57.7%)	376 (50.8%)	118 (59.6%)	
Male	4575 (52.5%)	3662 (54.9%)	469 (42.3%)	364 (49.2%)	80 (40.4%)	
Living area						0.240
Urban	3675 (42.2%)	2786 (41.8%)	484 (43.6%)	317 (42.8%)	88 (44.4%)	
Rural	5040 (57.8%)	3882 (58.2%)	625 (56.4%)	423 (57.2%)	110 (55.6%)	
Marital status						< 0.001
Married	3910 (44.9%)	3253 (48.8%)	272 (24.5%)	355 (48.0%)	30 (15.2%)	
Divorced, widowed or never married	4805 (55.1%)	3415 (51.2%)	837 (75.5%)	385 (52.0%)	168 (84.8%)	
Living arrangement						0.243
Alone	1473 (16.9%)	1154 (17.3%)	158 (14.2%)	133 (18.0%)	28 (14.1%)	
With family/caregivers	7242 (83.1%)	5514 (82.7%)	951 (85.8%)	607 (82.0%)	170 (85.9%)	
Education level						< 0.001
Illiterate	4427 (50.8%)	3177 (47.6%)	726 (65.5%)	392 (53.0%)	132 (66.7%)	
Primary	3125 (35.8%)	2594 (38.9%)	307 (27.6%)	176 (23.8%)	48 (24.2%)	
Secondary and above	1163 (13.4%)	897 (13.5%)	76 (6.9%)	172 (23.2%)	18 (9.1%)	
Multimorbidity^a^						0.102
Yes	2154 (24.7%)	1592 (23.9%)	342 (30.8%)	167 (22.6%)	53 (26.8%)	
No	6561 (75.3%)	5076 (76.1%)	767 (69.2%)	573 (77.4%)	145 (73.2%)	
Household income^b^						0.489
< Median income	3993 (45.8%)	3074 (46.1%)	456 (41.1%)	363 (49.1%)	100 (50.5%)	
≥ Median income	4722 (54.2%)	3594 (53.9%)	653 (58.9%)	377 (50.9%)	98 (49.5%)	
Preference for institutional care^c^						0.396
Yes	102 (1.2%)	83 (1.2%)	9 (0.8%)	8 (1.1%)	2 (1.0%)	
No	8613 (98.8%)	6585 (98.8%)	1100 (99.2%)	732 (98.9%)	196 (99.0%)	
Follow‐up status in 2018						< 0.001
TIC	105 (1.2%)	79 (1.2%)	11 (1.0%)	9 (1.2%)	6 (3.0%)	
Death	4108 (47.1%)	2914 (43.7%)	746 (67.3%)	320 (43.1%)	128 (64.7%)	
Lost to follow‐up	2793 (32.1%)	2198 (33.0%)	302 (27.2%)	234 (31.6%)	59 (29.8%)	
No event	1709 (19.6%)	1477 (22.1%)	50 (4.5%)	177 (23.9%)	5 (2.5%)	
Incidence of TIC (per 1000 person‐years)	2.3 (1.9–2.8)	2.1 (1.7–2.7)	2.8 (1.5–5.2)	2.2 (1.1–4.4)	9.6 (3.9–21.9)	—

^a^ For multimorbidity, participants with two or more self‐reported chronic diseases were considered ‘yes’, otherwise ‘no’. Chronic diseases considered in this study included hypertension, diabetes, heart disease, stroke, bronchitis, tuberculosis, cataract, glaucoma, cancer, gastric ulcer, Parkinson's disease, arthritis, epilepsy, cholecystitis, blood disease, chronic nephritis and hepatitis.

^b^ For household income, participants below the median household income for the living area (East, Central and West) were considered ‘< median income’, otherwise ‘≥ median income’.

^c^ For preference for institutional care, participants answered “institution” to the question “what kind of living arrangement do you like best?” were considered as yes, while those answered “living alone (or with spouse)” or “co‐residence with children” were considered as no.

Abbreviations: CF: cognitive frailty; TIC: transition to institutional care.

During the mean of 5.3 years (SD = 3.4) with 45,898 person‐years of follow‐up, 1.2% (95% CI: 1.0%–1.4%) transitioned to institutional care, 47.1% (95% CI: 46.1%–48.2%) died before TIC and 32.1% (95% CI: 31.1%–33.0%) were lost to follow‐up. Supporting Information S1: Table S2 shows the comparison of baseline characteristics between those lost to follow‐up and completers. The mean time to TIC was 3.7 years (SD = 2.7), with 4.0 (SD = 2.8) for unimpaired individuals, 3.0 (SD = 2.6) for those with physical frailty only, 3.0 (SD = 2.3) for those with cognitive impairment only, and 2.5 (SD = 1.6) for those with CF. The incidence rates of TIC, mortality and lost to follow‐up were 2.3 (95% CI: 1.9–2.8), 89.5 (95% CI: 86.9–92.2) and 60.8 (95% CI: 58.7–63.1) per 1000 person‐years. The incidence rate of TIC among people with CF was 9.6 (95% CI: 3.9–21.9) per 1000 person‐years.

### 3.2 Subdistribution Hazards Model for TIC on CF

CF was associated with a greater hazard of TIC (SHR 3.51, 95% CI: 1.49 to 8.28; *p* = 0.004) compared to the unimpaired group. Additionally, a higher risk of TIC was observed among individuals aged 75–84 years (SHR 1.91, 95% CI: 1.09 to 3.32; *p* = 0.023) and those aged ≥ 85 years (SHR 2.01, 95% CI: 1.12 to 3.62; *p* = 0.020), compared to those aged 65–74 years. Participants living in urban areas had a greater risk of TIC (SHR 2.48, 95% CI: 1.66 to 3.72; *p* < 0.001) than those in rural areas. Similarly, living alone was associated with a higher hazard of TIC (SHR 2.62, 95% CI: 1.58 to 4.36; *p* < 0.001) compared to living with family or caregivers. Participants who expressed a preference for institutional care also had an increased risk (SHR 6.76, 95% CI: 3.22 to 14.18; *p* < 0.001) compared to those without such a preference (Table 2).

**TABLE 2:** Subdistribution hazards model for TIC on CF.

Variables	Model 1	
	SHRs	*p*‐value
Frailty and cognitive impairment status (ref: unimpaired)		0.041
Physical frailty only	1.11 (0.59–2.17)	0.718
Cognitive impairment only	1.12 (0.56–2.24)	0.747
CF	3.51 (1.49–8.28)	0.004
Gender (ref: female)		
Male	1.03 (0.66–1.60)	0.906
Age (ref: 65–74)		0.043
75–84	1.91 (1.09–3.32)	0.023
≥ 85	2.01 (1.12–3.62)	0.020
Living area (ref: rural)		
Urban	2.48 (1.66–3.72)	< 0.001
Marital (ref: divorced, widowed or never married)		
Married	1.31 (0.80–2.29)	0.264
Living arrangement (ref: with family/caregivers)		
Alone	2.62 (1.58–4.36)	< 0.001
Educational level (ref: illiterate)		
Non‐literate	0.93 (0.60–1.45)	0.930
Multimorbidity (ref: no)^a^		
Yes	1.08 (0.70–1.68)	0.730
Household income (ref: < Median income)^b^		
≥ median income	0.80 (0.52–1.22)	0.290
Preference for institutional care (ref: no)^c^		
Yes	6.76 (3.22–14.18)	< 0.001

^a^ For multimorbidity, participants with two or more self‐reported chronic diseases were considered ‘yes’, otherwise ‘no’. Chronic diseases considered in this study included hypertension, diabetes, heart disease, stroke, bronchitis, tuberculosis, cataract, glaucoma, cancer, gastric ulcer, Parkinson's disease, arthritis, epilepsy, cholecystitis, blood disease, chronic nephritis and hepatitis.

^b^ For household income, participants below the median household income for the living area (East, Central and West) were considered ‘< median income’, otherwise ‘≥ median income’.

^c^ For preference for institutional care, participants answered “institution” to the question “what kind of living arrangement do you like best?” were considered as yes, while those answered “living alone (or with spouse)” or “co‐residence with children” were considered as no.

Abbreviations: CF: cognitive frailty; TIC: transition to institutional care; SHRs: subdistribution hazards ratios.

People with CF had a higher TIC risk than those with physical frailty only (SHR 3.15, 95% CI: 1.17–8.57; *p* = 0.024) and cognitive impairment only (SHR 3.14, 95% CI: 1.09–8.98; *p* = 0.034) (Table 3). The results of the sensitivity analyses were largely consistent (Supporting Information S1: Table S3‐S5). For urban‐rural disparities, due to the limited number of TIC cases (63 in urban areas and 42 in rural areas), only a subset of covariates could be included in the model. The results showed that CF was significantly associated with a higher risk of TIC (SHR 4.40, 95% CI: 1.57–12.33; *p* = 0.005) compared to unimpaired in urban areas, whereas the corresponding SHR in rural areas was 3.30 (95% CI: 0.79–13.85), statistical significance was not reached (*p* = 0.102) (Supporting Information S1: Table S6).

**TABLE 3:** Pairwise comparison of CF with different statuses based on subdistribution hazards model for TIC.

Groups	SHRs	*p*‐value
CF versus unimpaired	3.51 (1.49–8.28)	0.004
CF versus physical frailty only	3.15 (1.17–8.57)	0.024
CF versus cognitive impairment only	3.14 (1.09–8.98)	0.034

*Note:* With mortality and lost to follow‐up as two competing risks and adjusting for gender, age, living area, education level, marital status, living arrangement, multimorbidity, household income and preference for institutional care.

Abbreviations: CF: cognitive frailty; SHRs: subdistribution hazards ratios.

### 3.3 Subdistribution Hazards Models for Mortality and Lost to Follow‐Up

CF was associated with a higher risk of mortality (SHR 1.50, 95% CI: 1.25–1.79; *p* < 0.001) and lost to follow‐up (SHR 1.38, 95% CI: 1.06–1.80; *p* = 0.016) compared with the unimpaired group (Table 4). Full subdistribution hazards models are presented in Supporting Information S1: Table S7.

**TABLE 4:** Subdistribution hazards models for mortality and lost to follow‐up.

Variables	Model 2 (mortality)		Model 3 (lost to follow‐up)	
	SHRs	*p*‐value	SHRs	*p*‐value
Frailty and cognitive impairment status (ref: unimpaired)		< 0.001		0.038
Physical frailty only	1.49 (1.37–1.63)	< 0.001	1.13 (0.99–1.28)	0.068
Cognitive impairment only	1.09 (0.97–1.23)	0.134	1.00 (0.87–1.14)	0.983
CF	1.50 (1.25–1.79)	< 0.001	1.38 (1.06–1.80)	0.016

*Note:* Model 2 with TIC and lost to follow‐up as two competing risks, and Model 3 with mortality and TIC as two competing risks. Both models adjusting for gender, age, living area, education level, marital status, living arrangement, multimorbidity, household income and preference for institutional care.

Abbreviations: CF: cognitive frailty; SHRs: subdistribution hazards ratios.

## 4 Discussion

To the best of our knowledge, this is the first study to explore the association between CF and TIC among community‐dwelling older adults using a longitudinal dataset. By using the subdistribution hazards model with mortality and lost to follow‐up as two competing events, our findings demonstrated that CF was significantly associated with an increased risk of TIC.

In our sample of older adults without dementia, the prevalence of CF was 2.3% (95% CI: 2.0%–2.6%), which falls within the previously reported range of 1.6%–35.7% among Chinese community‐dwelling older adults [35, 36]. Among these, except for three studies which reported prevalence rates of CF at 1.6% in 2011 [36], 2.3% in 2011 [37], and 4.2% in 2014–2016 [38], all other studies included in the review were conducted after 2017. The prevalence of CF increased year by year [15], which may explain the lower prevalence observed in those studies. Furthermore, the low prevalence may be influenced by the exclusion of participants with missing data on CF, which is unavoidable in large‐scale epidemiological investigations [36]. For example, participants who were very frail were often unable to complete the MMSE and were therefore more likely to be excluded. Variation in prevalence across studies may also result from differences in baseline populations, particularly the inclusion [36], or exclusion [37] of individuals with dementia. Additionally, unlike earlier studies that only excluded participants with self‐reported dementia [39], our study also excluded those with cognitive test‐based dementia. This decision reflects the possibility that some individuals, particularly rural residents, may not have received a formal diagnosis of dementia due to limited access to healthcare or lack of awareness. These differences in exclusion criteria may partly explain the variation in CF prevalence observed across studies.

Moreover, we found people with CF had a higher TIC risk than those unimpaired, physical frailty only and cognitive impairment only. Our findings showed that in addition to the well‐studied adverse health outcomes of disability, fall and poor higher‐level competence [16, 40], TIC also demonstrated similar associations with CF, that is, the elevated risk of the negative health effects of CF may be more severe than physical frailty only or cognitive impairment only. In terms of the strength of association between CF and long‐term care needs, a 2‐year follow‐up study in Japan reported that the risk of the onset of long‐term care needs for people with CF was 3.86 times (95% CI, 2.95–5.05) that of robust individuals [21]. Similarly, a study in South Korea found that CF was associated with a higher risk of long‐term care needs than robust individuals (HR, 2.65; 95% CI, 2.08–3.35) [20]. While our study targeted at the actual utilisation of institutional care, we reported a SHR of 3.51 when comparing CF to unimpaired, indicating a similar magnitude of risk to studies assessing long‐term care needs. Moreover, we observed that CF was significantly associated with a higher risk of mortality, consistent with a recent review that reported a HR of 2.01 (95% CI, 1.84–2.19) [41]. Cognitive impairment and physical frailty have been identified as two independent predictors of attrition [42], and our study further corroborated the relationship between CF and lost to follow‐up.

We adjusted all possible factors in the model based on previous studies [18], available data and modelling requirements. Among them, advanced age, living areas, living arrangement and preference for institutional care were significantly associated with an increased risk of TIC. Our study showed that advanced age was a significant predictor of TIC, consistent with previous research [43]. The hazard of TIC among urban residents was more than twice that of rural residents, consistent with previous studies [9, 44]. This relationship was significant in urban areas but not in rural areas in the preliminary stratified analysis. One possible explanation is that supportive household structures are more common in rural areas than in urban areas, which may provide rural residents with more informal support [44]. Additionally, institutional care is more accessible to urban residents, further leading to urban‐rural differences in TIC [45, 46]. However, in the stratified analysis, the lack of statistical significance in rural areas should be interpreted with caution, as the relatively small number of events in this subgroup likely limited the power to detect meaningful differences. Therefore, the absence of significant findings does not necessarily imply the absence of meaningful disparities in rural areas. Future research with larger rural samples and longer follow‐up periods is needed to clarify these disparities and inform targeted strategies.

Regarding living arrangement, our findings indicated that living alone was a strong predictor of TIC, consistent with previous studies [19, 47]. While the majority of older adults prefer to age in place, declining in health condition and physical function often necessitate external support, and inadequate support may result in nursing home admission [48]. Living alone may also reflect social isolation and limited access to informal caregiving, thereby increasing TIC risk [49]. Moreover, consistent with prior findings [19], a preference for institutional care was positively associated with TIC. This may be because older adults with such preferences often hold positive attitudes towards institutional care and are more likely to proactively plan for admission.

In this study, the incidence of TIC was 2.3 (95% CI: 1.9–2.8) per 1000 person‐years, lower than the incidence of 3.1 (95% CI: 2.9–3.4) per 1000 person‐years in South Korea [20]. A similar situation was observed for institutionalisation rates. Our study reported a 1.2% rate over 5.3 years, compared with 0.8% at 3 years in mainland China [9], 2.8% at 3 years in Hong Kong [50], 3.4% at 1 year in Taiwan [51], and 17.3% at 3.3 years in South Korea [52]. The lower rate in our study may be due to the higher rate of lost to follow‐up (32.1%) over the 10‐year period, the exclusion of individuals with dementia and the characteristics of the study population. Our study participants were also older (sample mean age of 82.1 years), whereas TIC may have occurred at younger ages. Additionally, the older sample may result in a higher incidence of death, further affecting the incidence of TIC.

Given the higher risk of TIC associated with CF, interventions should be implemented to delay premature progression to CF, or even to reverse it where possible. Ruan et al. [22] proposed a three‐tiered preventive intervention framework for different stages of CF including physical, nutritional, cognitive, and psychological domains, to reduce the risk of developing CF and the occurrence of CF‐related adverse outcomes. A recent multidomain intervention for the reversal of CF among multi‐ethnic older adults in Malaysia showed positive preliminary results at 12 months [53]. Additionally, a meta‐analysis of 11 trials conducted in China, Singapore, Spain, the United States and Austria indicated that multicomponent exercises improved cognitive function and frailty status for people with CF [54].

Greater emphasis should also be placed on older adults living alone by providing them with appropriate community and social support to ensure timely access to assistance when needed, thereby reducing the risk of premature institutionalisation. Furthermore, tailored interventions and appropriate resource allocation should be developed to address urban‐rural disparities, which may be influenced by multiple factors such as the level of economic development, service availability, and care preferences [55]. Additionally, the stratified analysis suggests that rural areas with lower TIC, although not statistically significant due to the small number of TIC cases, may reflect populations that are more vulnerable or have less access to formal care services. Therefore, broader policy implications should consider potential imbalances between care demand and supply, which warrant further investigation from both perspectives to inform more equitable and effective strategies. Moreover, care planning should incorporate assessments of individual preferences and the broader social context, ensuring that long‐term care decisions are both person‐centred and culturally responsive. Policymakers should also take these preferences into account when designing long‐term care services and allocating resources, thereby creating care systems that are truly responsive and tailored to the diverse needs of the ageing population.

The strength of this study lies in the use of a nationally representative cohort database, which enhances the generalisability of the findings. Furthermore, the inclusion of 10‐year follow‐up data allowed sufficient time to capture the occurrence of various events of interest. Moreover, mortality and lost to follow‐up were treated as competing events in the analysis of TIC, thereby reducing potential bias in the estimates. Nevertheless, there are several limitations. First, while time‐to‐event analysis appropriately accounts for censoring and competing risks, the limited granularity of timing data may affect the precision of the estimated associations. Future studies should collect data on the actual timing of transitions to provide more accurate estimates. Second, TIC does not necessarily imply poorer health status, as it may also reflect differences in service availability or socioeconomic factors. However, due to data limitations, we were unable to account for all possible confounders, particularly contextual factors such as the availability of institutional care and other long‐term care services. Third, despite applying the Fine‐Gray subdistribution hazard model and treating lost to follow‐up as a competing event, the relatively high attrition rate may introduce potential bias. Lastly, subject to the observational feature, no causal conclusion could be made in the present study.

## 5 Conclusions

Older adults with CF were at a higher risk of TIC. The findings suggest that policies and interventions are needed to improve or maintain the physical and cognitive functions of older adults, thereby delaying premature institutionalisation and improving quality of life. Emphasis should also be placed on older adults living alone to ensure that they have timely access to assistance when needed.

## Funding

This research did not receive any specific grant from funding agencies in the public, commercial, or not‐for‐profit sectors.

## Conflicts of Interest

The authors declare no conflicts of interest.

## Supporting information


Supporting Information S1

## Data Availability

The data that support the findings of this study are openly available in Peking University Open Research Data Platform at https://opendata.pku.edu.cn.
