# Supplementary material for: Increased Risk of Transition to Institutional Care Among Community‐Dwelling Older Adults With Cognitive Frailty: A Competing Risks Survival Analysis
Source: Int J Geriatr Psychiatry. 2026 Feb 3;41(2):e70197. doi: 10.1002/gps.70197 (PMC12869130; doi:10.1002/gps.70197)
Supplement: Supplementary file 1 — Supporting Information S1 [file GPS-41-e70197-s001.docx]

**Supplemental Material**

**Increased risk of transition to institutional care among community-dwelling older adults with cognitive frailty: A competing risks survival analysis**

**Table S1.** The operational definition of CF in this study

**Text S1.** Assessment of intra-class correlation across provinces

**Figure S1.** Flowchart for study sample selection

**Table S2.** Baseline characteristics of participants lost to follow-up and completers

**Table S3.** Baseline characteristics of participants with and without missing data on CF status

**Table S4.** Sensitivity analyses

**Table S5.** Pairwise comparisons with different statuses in sensitivity analyses of models for TIC

**Table S6.** Subdistribution hazards model for TIC on CF in urban (N=3675) and rural areas (N=5040)

**Table S7.** Subdistribution hazards models for mortality and lost to follow-up

**Table S1.** The operational definition of CF in this study

|  | **Measurements** | **Cutoffs** |
| --- | --- | --- |
| Physical frailty | Exhaustion [3]: Participants answered ‘always’, ‘often’ or ‘sometimes’ to the question ‘I felt old and useless’. | Participants meeting ≥3 domains were defined as physical frailty [4]. |
|  | Shrink [4]: Body mass index < 18.5 kg/m2. |  |
|  | Weakness [5]: Participants’ self-reported inability to lift a bag weighing 5 kg |  |
|  | Low mobility [6]: Participants’ self-reported inability to walk 1 km. |  |
|  | Inactivity [7]: Participants reported that they engaged in once a week or less of the following light physical activities, including housework, outside activity, gardening, keeping a pet, livestock breeding, playing cards or mah-jong, and social activity. |  |
| Cognitive impairment and dementia | The MMSE-C included seven cognitive function tests, namely orientation, naming, registration, attention and calculation, copying a figure, recalling, and language capability [8]. The scores of the MMSE-C ranged between 0 and 30, with low scores indicating poor cognitive function. | The optimal cut-off scores for the groups aged ≤ 75 years old and education ≤ 6 years, aged > 75 years old and education ≤ 6 years, aged ≤ 75 years old and education > 6 years, aged > 75 years old and education > 6 years in screening for mild cognitive impairment and dementia were 26.5, 22.5, 28.5 and 26.5, and 23.5, 19.5, 23.5 and 23.5, respectively [9]. |
| Abbreviations: CF: cognitive frailty; MMSE-C: Chinese version of Mini-Mental Status Examination | | |

**Text S1.** Assessment of intra-class correlation across provinces

To assess potential province‑level clustering, we fitted a null multilevel logistic regression model with province-level random intercepts. The intra-class correlation coefficient (ICC) was 0.036, indicating that approximately 3.6% of the variance in TIC was attributable to differences between provinces. Given the modest magnitude of this clustering effect (ICC < 0.05) and the limited number of observations per province, we opted not to incorporate a full multilevel modelling structure. Instead, we used stratified analyses by urban-rural status, which aligns with our study objectives and provides clearer interpretability in the context of structural disparities commonly observed between urban and rural areas.

**Figure S1.** Flowchart for study sample selection


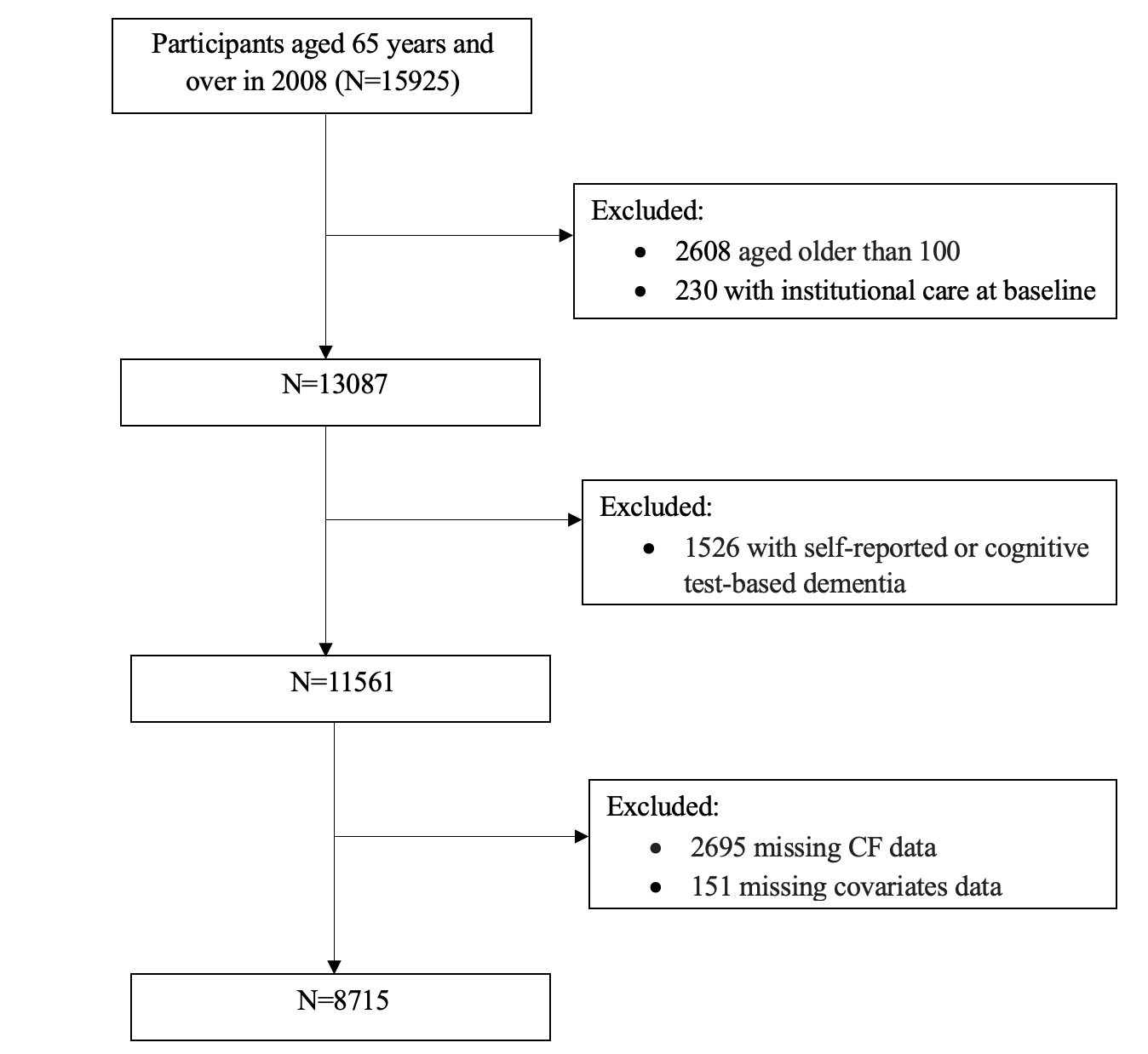


Table S2 summarises and compares the baseline characteristics of participants lost to follow‑up and completers. Significant differences were observed across all variables (except for preference for institutional care) despite the small magnitude of difference in most of the cases (P<0.05).

**Table S2.** Baseline characteristics of participants lost to follow-up and completers

| **Variables** | **All** | **Lost to follow-up** | **Not lost to follow-up** | **P-value** |
| --- | --- | --- | --- | --- |
| No. of participants | 8715 | 2793 (32.1%) | 5922 (67.9%) | - |
| Age (mean±SD) | **82.1±9.5** | **80.6±9.7** | **82.8±9.4** | <0.001 |
| 65-74 | 2326 (26.7%) | 898 (32.2%) | 1428 (24.1%) |  |
| 75-84 | 2590 (29.7%) | 845 (30.3%) | 1745 (29.5%) |  |
| ≥85 | 3799 (43.6%) | 1050 (37.6%) | 2749 (46.4%) |  |
| Gender |  |  |  | <0.001 |
| Female | 4140 (47.5%) | 1411 (50.5%) | 2729 (46.1%) |  |
| Male | 4575 (52.5%) | 1382 (49.5%) | 3193 (53.9%) |  |
| Living areas |  |  |  | <0.001 |
| Urban | 3675 (42.2%) | 1552 (55.6%) | 2123 (35.9%) |  |
| Rural | 5040 (57.8%) | 1241 (44.4%) | 3799 (64.1%) |  |
| Marital status |  |  |  | <0.001 |
| Married | 3910 (44.9%) | 1330 (47.3%) | 2580 (43.6%) |  |
| Divorced, widowed or never married | 4805 (55.1%) | 1463 (52.4%) | 3342 (56.4%) |  |
| Living arrangement |  |  |  | 0.032 |
| Alone | 1473 (16.9%) | 507 (18.2%) | 966 (16.3%) |  |
| With family/caregivers | 7242 (83.1%) | 2286 (81.8%) | 4956 (83.7%) |  |
| Education level |  |  |  | <0.001 |
| Illiterate | 4427 (50.8%) | 1286 (46.0%) | 3141 (53.0%) |  |
| Primary | 3125 (35.8%) | 1010 (36.2%) | 2115 (35.7%) |  |
| Secondary and above | 1163 (13.4%) | 497 (17.8%) | 666 (11.2%) |  |
| Multimorbidity ^a^ |  |  |  | <0.001 |
| Yes | 2154 (24.7%) | 776 (27.8%) | 1378 (23.3%) |  |
| No | 6561 (75.3%) | 2017 (72.2%) | 4544 (76.7%) |  |
| Household income ^b^ |  |  |  | <0.001 |
| < Median income | 3993 (45.8%) | 1059 (37.9%) | 2934 (49.5%) |  |
| ≥ Median income | 4722 (54.2%) | 1734 (62.1%) | 2988 (50.5%) |  |
| Preference for institutional care ^c^ | |  |  | 0.119 |
| Yes | 102 (1.2%) | 40 (1.4%) | 62 (1.0%) |  |
| No | 8613 (98.8%) | 2753 (98.6%) | 5860 (99.0%) |  |
| Frailty and cognitive impairment status | |  |  | 0.002 |
| Unimpaired | 6668 (76.5%) | 2198 (78.7%) | 4470 (75.5%) |  |
| Physical frailty only | 1109 (12.7%) | 302 (10.8%) | 807 (13.6%) |  |
| Cognitive impairment only | 740 (8.5%) | 234 (8.4%) | 506 (8.6%) |  |
| CF | 198 (2.3%) | 59 (2.1%) | 139 (2.3%) |  |
| Note: ^a^ For multimorbidity, participants with two or more self-reported chronic diseases were considered ‘yes’, otherwise ‘no’. Chronic diseases considered in this study included hypertension, diabetes, heart disease, stroke, bronchitis, tuberculosis, cataract, glaucoma, cancer, gastric ulcer, Parkinson’s disease, arthritis, epilepsy, cholecystitis, blood disease, chronic nephritis and hepatitis.  ^b^ For household income, participants below the median household income for the living area (East, Central and West) were considered ‘< median income’, otherwise ‘≥ median income’. | | | | |

To address potential bias due to missing data, we conducted a sensitivity analysis in which missing data on CF status and covariates were handled using multiple imputation.

We first assessed the missingness patterns and found that missingness in covariates could reasonably be assumed to be missing at random (MAR), whereas missingness in CF status was not missing at random. We further examined factors associated with missing CF status and identified that age, gender, living area, marital status, education level, and household income were significantly associated with missingness (see Supplementary Table S3).

Multiple imputation was then applied to both CF status and covariates using the identified associated variables. The results of this sensitivity analysis were largely consistent with those of the main analysis (Supplementary Tables S4 and S5).

**Table S3.** Baseline characteristics of participants with and without missing data on CF status

| **Variables** | **All** | **With missing data on CF status** | **Without missing data on CF status** | **P-value** |
| --- | --- | --- | --- | --- |
| No. of participants | 11561 | 2695 (23.3%) | 8866 (76.7%) | - |
| Age (mean±SD) | **84.0±9.8** | **90.5±7.9** | **82.1±9.5** | <0.001 |
| 65-74 | 2549 (22.1%) | 190 (7.1%) | 2359 (26.6%) |  |
| 75-84 | 2937 (25.4%) | 307 (11.3%) | 2630 (29.7%) |  |
| ≥85 | 6075 (52.5%) | 2198 (81.6%) | 3877 (43.7%) |  |
| Gender |  |  |  | <0.001 |
| Female | 5967 (51.6%) | 1749 (64.9%) | 4218 (47.6%) |  |
| Male | 5594 (48.4%) | 946 (35.1%) | 4648 (52.4%) |  |
| Living areas |  |  |  | <0.001 |
| Urban | 4695 (40.6%) | 967 (35.9%) | 3728 (42.0%) |  |
| Rural | 6866 (59.4%) | 1728 (64.1%) | 5138 (58.0%) |  |
| Marital status |  |  |  | <0.001 |
| Married | 4500 (38.9%) | 554 (20.6%) | 3946 (44.5%) |  |
| Divorced, widowed or never married | 7061 (61.1%) | 2141 (79.4%) | 4920 (55.5%) |  |
| Living arrangement |  |  |  | 0.673 |
| Alone | 1998 (17.3%) | 473 (17.6%) | 1525 (17.2%) |  |
| With family/caregivers | 9563 (82.7%) | 2222 (82.4%) | 7341 (82.8%) |  |
| Education level |  |  |  | <0.001 |
| Illiterate | 6570 (56.8%) | 2060 (76.5%) | 4510 (50.9%) |  |
| Primary | 3672 (31.8%) | 489 (18.1%) | 3183 (35.9%) |  |
| Secondary and above | 1291 (11.2%) | 118 (4.3%) | 1173 (13.2%) |  |
| Missing | 28 (0.2%) | 28 (1.0%) | - |  |
| Multimorbidity ^a^ |  |  |  | 0.131 |
| Yes | 2816 (24.4%) | 627 (23.3%) | 2189 (24.7%) |  |
| No | 8745 (75.6%) | 2068 (76.7%) | 6677 (75.3%) |  |
| Household income ^b^ |  |  |  | <0.001 |
| < Median income | 5546 (48.0%) | 1470 (54.5%) | 4076 (46.0%) |  |
| ≥ Median income | 6010 (51.9%) | 1223 (45.4%) | 4787 (53.9%) |  |
| Missing | 5 (0.1%) | 2 (0.1%) | 3 (0.1%) |  |
| Preference for institutional care ^c^ | |  |  | 0.417 |
| Yes | 135 (1.2%) | 33 (1.2%) | 102 (1.2%) |  |
| No | 10983 (95.0%) | 2367 (87.9%) | 8616 (97.1%) |  |
| Missing | 443 (3.8%) | 295 (10.9%) | 148 (1.7%) |  |
| Note: ^a^ For multimorbidity, participants with two or more self-reported chronic diseases were considered ‘yes’, otherwise ‘no’. Chronic diseases considered in this study included hypertension, diabetes, heart disease, stroke, bronchitis, tuberculosis, cataract, glaucoma, cancer, gastric ulcer, Parkinson’s disease, arthritis, epilepsy, cholecystitis, blood disease, chronic nephritis and hepatitis.  ^b^ For household income, participants below the median household income for the living area (East, Central and West) were considered ‘< median income’, otherwise ‘≥ median income’. | | | | |

Table S4 presents the results of sensitivity analyses. The results showed that cognitive frailty was associated with greater hazard for TIC compared to unimpaired when physical frailty was based on the Frailty Index instead of the Fried criteria (SHR 5.65, 95% CI: 1.76-18.17; p=0.004), when ‘not able to answer’ responses in MMSE-C was treated as incorrect answers (SHR 2.26, 95% CI: 1.10-4.65; p=0.027), when the time-to-event was defined as the interval from the baseline survey to the follow-up survey at which the first event occurred (SHR 3.48, 95% CI: 1.48-8.22; p=0.004), when participants lost to follow-up were censored (SHR 3.73, 95% CI: 1.58-8.80; p=0.003), and when missing data were imputed (SHR 2.62, 95% CI: 1.10-6.26; p=0.030).

**Table S4.** Sensitivity analyses

| **Models** | **Based on the Frailty Index and MMSE-C** | | **“Not able to answer” responses in MMSE-C as incorrect answers** | | **Time-to-event: baseline to first event follow-up** | | **Lost to follow-up was censored** | | **Missing data were imputed** | |  |  |  |
| --- | --- | --- | --- | --- | --- | --- | --- | --- | --- | --- | --- | --- | --- |
| **Variables** | **SHRs** | **P-value** | **SHRs** | **P-value** | **SHRs** | **P-value** | **SHRs** | **P-value** | **SHRs** | **P-value** |  |  |  |
| Frailty and Cognitive Impairment status (ref: unimpaired) | | **0.030** |  | 0.181 |  | **0.044** |  | **0.029** |  | 0.187 |  |  |  |
| Physical frailty only | 1.26 (0.51-3.12) | 0.619 | 1.13 (0.59-2.18) | 0.706 | 1.11 (0.59-2.17) | 0.745 | 1.14 (0.59-2.19) | 0.696 | 0.94 (0.48-1.86) | 0.846 |  |  |  |
| Cognitive impairment only | 1.30 (0.71-2.38) | 0.404 | 1.09 (0.62-1.94) | 0.762 | 1.12 (0.56-2.23) | 0.754 | 1.14 (0.57-2.29) | 0.704 | 1.10 (0.56-2.17) | 0.781 |  |  |  |
| Cognitive frailty | 5.65 (1.76-18.17) | **0.004** | 2.26 (1.10-4.65) | **0.027** | 3.48 (1.48-8.22) | **0.004** | 3.73 (1.58-8.80) | **0.003** | 2.62 (1.10-6.26) | **0.030** |  |  |  |
| Gender (ref: female) |  |  |  |  |  |  |  |  |  |  |  |  |  |
| Male | 1.01 (0.65-1.58) | 0.950 | 1.04 (0.67-1.60) | 0.876 | 1.03 (0.66-1.60) | 0.903 | 1.01 (0.64-1.56) | 0.990 | 1.06 (0.70-1.60) | 0.775 |  |  |  |
| Age (ref: 65-74) |  | **0.033** |  | **0.045** |  | **0.047** |  | **0.038** |  | 0.062 |  |  |  |
| 75-84 | 1.93 (1.11-3.36) | **0.021** | 1.90 (1.10-3.26) | **0.021** | 1.88 (1.08-3.29) | **0.025** | 1.91 (1.09-3.32) | **0.023** | 1.83 (1.09-3.08) | **0.023** |  |  |  |
| ≥85 | 2.07 (1.16-3.69) | **0.013** | 1.93 (1.09-3.43) | **0.025** | 1.96 (1.10-3.53) | **0.022** | 2.06 (1.14-3.70) | **0.016** | 1.62 (0.94-2.82) | 0.084 |  |  |  |
| Living areas (ref: rural) |  |  |  |  |  |  |  |  |  |  |  |  |  |
| Urban | 2.55 (1.70-3.82) | **<0.001** | 2.63 (1.78-3.91) | **<0.001** | 2.45 (1.63-3.68) | **<0.001** | 2.71 (1.81-4.06) | **<0.001** | 2.70 (1.85-3.93) | **<0.001** |  |  |  |
| Marital (ref: divorced, widowed or never married) | |  |  |  |  |  |  |  |  |  |  |  |  |
| Married | 1.30 (0.77-2.19) | 0.334 | 1.15 (0.69-1.90) | 0.599 | 1.31 (0.76-2.22) | 0.215 | 1.32 (0.78-2.22) | 0.306 | 1.11 (0.69-1.81) | 0.663 |  |  |  |
| Living arrangement (ref: with family/caregivers) | | |  |  |  |  |  |  |  |  |  |  |  |
| Alone | 2.64 (1.58-4.41) | **<0.001** | 2.31 (1.42-3.76) | **<0.001** | 2.62 (1.57-4.35) | **<0.001** | 2.76 (1.66-4.61) | **<0.001** | 2.34 (1.49-3.70) | **<0.001** |  |  |  |
| Educational level (ref: illiterate) |  |  |  |  |  |  |  |  |  |  |  |  |  |
| Non-literate | 0.91 (0.58-1.41) | 0.668 | 1.05 (0.68-1.63) | 0.817 | 0.93 (0.60-1.45) | 0.750 | 0.97 (0.62-1.51) | 0.888 | 1.00 (0.66-1.52) | 0.995 |  |  |  |
| Multimorbidity (ref: no) ^a, #^ |  |  |  |  |  |  |  |  |  |  |  |  |  |
| Yes | - | - | 1.02 (0.67-1.57) | 0.915 | 1.08 (0.70-1.68) | 0.731 | 1.10 (0.71-1.71) | 0.973 | 1.03 (0.68-1.55) | 0.890 |  |  |  |
| Household income (ref: < median income) ^b^ | |  |  |  |  |  |  |  |  |  |  | | |
| ≥ Median income | 0.77 (0.50-1.18) | 0.223 | 0.77 (0.51-1.17) | 0.216 | 0.80 (0.52-1.22) | 0.289 | 0.85 (0.55-1.29) | 0.450 | 0.79 (0.54-1.16) | 0.233 |  |  |  |
| Preference for institutional care (ref: no) ^c^ | |  |  |  |  |  |  |  |  |  |  |  |  |
| Yes | 7.05 (3.36-14.80) | **<0.001** | 6.24 (2.99-13.03) | **<0.001** | 6.82 (3.23-14.31) | **<0.001** | 7.38 (3.52-15.49) | **<0.001** | 5.27 (2.54-10.96) | **<0.001** |  |  |  |
| Note: ^a^ For multimorbidity, participants with two or more self-reported chronic diseases were considered ‘yes’, otherwise ‘no’. Chronic diseases considered in this study included hypertension, diabetes, heart disease, stroke, bronchitis, tuberculosis, cataract, glaucoma, cancer, gastric ulcer, Parkinson’s disease, arthritis, epilepsy, cholecystitis, blood disease, chronic nephritis and hepatitis.  ^b^ For household income, participants below the median household income for the living area (East, Central and West) were considered ‘< median income’, otherwise ‘≥ median income’.  ^c^ For preference for institutional care, participants answered “institution” to the question “what kind of living arrangement do you like best?” were considered as yes, while those answered “living alone (or with spouse)” or “co-residence with children” were considered as no.  ^#^ As multimorbidity-related items were assessed in Frailty Index, multimorbidity was not treated as a covariate in sensitivity analyse when physical frailty was assessed based on the Frailty Index.  Abbreviations: MMSE-C: Chinese version of Mini-Mental Status Examination; SHRs: subdistribution hazards ratios | | | | | | | | | | |  |  |  |

Table S5 presents the SHRs for pairwise comparisons with different frailty and cognitive impairment statuses in sensitivity analyses.

**Table S5.** Pairwise comparisons with different statuses in sensitivity analyses of models for TIC

| **Variables** | **Based on the Frailty Index and MMSE-C** | | **“Not able to answer” responses as incorrect answers** | | **Time-to-event: baseline to first event follow-up** | | **Lost to follow-up was censored** | | **Missing data were imputed** | |
| --- | --- | --- | --- | --- | --- | --- | --- | --- | --- | --- |
|  | **SHRs** | **P-value** | **SHRs** | **P-value** | **SHRs** | **P-value** | **SHRs** | **P-value** | **SHRs** | **P-value** |
| Cognitive frailty vs Unimpaired | 5.65 (1.76-18.17) | **0.004** | 2.26 (1.10-4.65) | **0.027** | 3.48 (1.48-8.22) | **0.004** | 3.73 (1.58-8.80) | **0.003** | 2.62 (1.10-6.26) | **0.030** |
| Cognitive frailty vs Physical frailty only | 4.49 (1.07-18.85) | **0.040** | 1.99 (0.82-4.83) | 0.128 | 3.13 (1.15-8.49) | **0.025** | 3.27 (1.20-8.89) | **0.020** | 2.91 (1.05-8.35) | **0.041** |
| Cognitive frailty vs Cognitive impairment only | 4.36 (1.22-15.67) | **0.024** | 2.07 (0.88-4.87) | 0.097 | 3.12 (1.09-8.96) | **0.034** | 3.25 (1.13-9.37) | **0.029** | 2.47 (0.71-8.69) | 0.155 |
| Note: With mortality and lost to follow-up as two competing risks and adjusting for gender, age, living areas, education level, marital status, living arrangement, multimorbidity, household income and preference for institutional care.  Abbreviations: MMSE-C: Chinese version of Mini-Mental Status Examination; SHRs: subdistribution hazards ratios | | | | | | | | | | |

Table S6 presents the subdistribution hazards ratios (SHRs) and 95% confidence intervals (CIs) for TIC on CF in urban area. Due to sample limitation (with 63 urban residents and 42 rural residents transitioned to institutional care), not all covariates were included in the analysis. Cognitive frailty was associated with a higher risk of TIC (SHR 4.40, 95% CI: 1.57-12.33; p=0.005) in urban area compared to unimpaired.

**Table S6.** Subdistribution hazards model for TIC on CF in urban (N=3675) and rural areas (N=5040)

| **Variables** | **Urban** | | **Rural** | |
| --- | --- | --- | --- | --- |
|  | **SHRs** | **p value** | **SHRs** | **p value** |
| Frailty and Cognitive Impairment status (ref: unimpaired) | | 0.047 |  | 0.374 |
| Physical frailty only | 1.13 (0.48-2.66) | 0.781 | 1.40 (0.54-3.62) | 0.488 |
| Cognitive impairment only | 1.18 (0.50-2.76) | 0.703 | 0.87 (0.27-2.85) | 0.821 |
| CF | 4.40 (1.57-12.33) | 0.005 | 3.30 (0.79-13.85) | 0.102 |
| Living arrangement (ref: with family/caregivers) | |  |  |  |
| Alone | 3.07 (1.82-5.18) | <0.001 | 2.42 (1.27-4.59) | 0.007 |
| Preference for institutional care (ref: no) | | |  |  |
| Yes | 6.56 (2.31-18.58) | <0.001 | - | - |
| Abbreviations: CF: cognitive frailty; TIC: transition to institutional care; SHRs: subdistribution hazards ratios | | | | |

Table S7 presents the subdistribution hazards ratios (SHRs) and 95% confidence intervals (CIs) for mortality and lost to follow-up on cognitive frailty based on the competing risk regression models. Cognitive frailty was associated with a higher risk of mortality (SHR 1.50, 95% CI: 1.25-1.79; p<0.001) and lost to follow-up (SHR 1.38, 95% CI: 1.06-1.80; p=0.016) compared to unimpaired.

**Table S7.** Subdistribution hazards models for mortality and lost to follow-up

| **Variables** | **Model 2 (mortality)** | | **Model 3 (lost to follow-up)** | |
| --- | --- | --- | --- | --- |
|  | **SHRs** | **P-value** | **SHRs** | **P-value** |
| Frailty and Cognitive Impairment status (ref: unimpaired) | | <0.001 |  | 0.038 |
| Physical frailty only | 1.49 (1.37-1.63) | <0.001 | 1.13 (0.99-1.28) | 0.068 |
| Cognitive impairment only | 1.09 (0.97-1.23) | 0.134 | 1.00 (0.87-1.14) | 0.983 |
| Cognitive frailty | 1.50 (1.25-1.79) | <0.001 | 1.38 (1.06-1.80) | 0.016 |
| Gender (ref: female) |  |  |  |  |
| Male | 1.45 (1.35-1.56) | <0.001 | 0.91 (0.84-0.99) | 0.032 |
| Age (ref: 65-74) |  | <0.001 |  | 0.093 |
| 75-84 | 2.25 (2.01-2.51) | <0.001 | 0.99 (0.90-1.10) | 0.885 |
| ≥85 | 4.24 (3.80-4.73) | <0.001 | 1.10 (0.99-1.22) | 0.084 |
| Living area (ref: rural) |  |  |  |  |
| Urban | 0.68 (0.60-0.77) | <0.001 | 2.01 (1.74-2.33) | <0.001 |
| Living area*log(t) | 1.19 (1.08-1.30) | <0.001 | 0.87 (0.79-0.96) | 0.006 |
| Marital (ref: divorced, widowed or never married) | |  |  |  |
| Married | 0.78 (0.72-0.84) | <0.001 | 1.01 (0.92-1.11) | 0.822 |
| Living arrangement (ref: with family/caregivers) | |  |  |  |
| Alone | 0.84 (0.77-0.92) | <0.001 | 1.57 (1.31-1.89) | <0.001 |
| Alone*log(t) |  |  | 0.85 (0.74-0.97) | 0.013 |
| Educational level (ref: Illiterate) |  |  |  |  |
| Non-literate | 0.91 (0.85-0.98) | 0.009 | 1.09 (1.00-1.19) | 0.059 |
| Multimorbidity (ref: no) ^a^ |  |  |  |  |
| Yes | 1.02 (0.95-1.10) | 0.566 | 1.06 (0.98-1.16) | 0.151 |
| Household income (ref: < median income) ^b^ |  |  |  |  |
| ≥ Median income | 0.97 (0.91-1.04) | 0.340 | 1.76 (1.51-2.06) | <0.001 |
| Household income*log(t) |  |  | 0.81 (0.73-0.90) | <0.001 |
| Preference for institutional care (ref: no) |  |  |  |  |
| Yes | 1.10 (0.81-1.48) | 0.551 | 1.62 (1.18-2.21) | 0.003 |
| Note: ^a^ For multimorbidity, participants with two or more self-reported chronic diseases were considered ‘yes’, otherwise ‘no’. Chronic diseases considered in this study included hypertension, diabetes, heart disease, stroke, bronchitis, tuberculosis, cataract, glaucoma, cancer, gastric ulcer, Parkinson’s disease, arthritis, epilepsy, cholecystitis, blood disease, chronic nephritis and hepatitis.  ^b^ For household income, participants below the median household income for the living area (East, Central and West) were considered ‘< median income’, otherwise ‘≥ median income’.  ^c^ For preference for institutional care, participants answered “institution” to the question “what kind of living arrangement do you like best?” were considered as yes, while those answered “living alone (or with spouse)” or “co-residence with children” were considered as no.  Abbreviations: SHRs: subdistribution hazards ratios | | | | |

**References**

1. Austin, P.C., D.S. Lee, and J.P. Fine, *Introduction to the Analysis of Survival Data in the Presence of Competing Risks.* Circulation, 2016. **133**(6): p. 601-9. <https://doi.org/10.1161/circulationaha.115.017719>

2. Latouche, A., A. Allignol, J. Beyersmann, M. Labopin, and J.P. Fine, *A competing risks analysis should report results on all cause-specific hazards and cumulative incidence functions.* J Clin Epidemiol, 2013. **66**(6): p. 648-53. <https://doi.org/10.1016/j.jclinepi.2012.09.017>

3. Huang, J., X. Zeng, H. Ning, et al., *Development and validation of prediction model for older adults with cognitive frailty.* Aging Clin Exp Res, 2024. **36**(1): p. 8. <https://doi.org/10.1007/s40520-023-02647-w>

4. Wang, H.Y., X. Lv, J. Du, G. Kong, and L. Zhang, *Age- and Gender-Specific Prevalence of Frailty and Its Outcomes in the Longevous Population: The Chinese Longitudinal Healthy Longevity Study.* Front Med (Lausanne), 2021. **8**: p. 719806. <https://doi.org/10.3389/fmed.2021.719806>

5. Singh, S., K.R. Bailey, A. Noheria, and I.J. Kullo, *Frailty Across the Spectrum of Ankle-Brachial Index.* Angiology, 2012. **63**(3): p. 229-236. <https://doi.org/10.1177/0003319711413457>

6. van Kan, G.A., Y.M. Rolland, J.E. Morley, and B. Vellas, *Frailty: Toward a Clinical Definition.* Journal of the American Medical Directors Association, 2008. **9**(2): p. 71-72. <https://doi.org/10.1016/j.jamda.2007.11.005>

7. Hanlon, P., B.I. Nicholl, B.D. Jani, et al., *Frailty and pre-frailty in middle-aged and older adults and its association with multimorbidity and mortality: a prospective analysis of 493 737 UK Biobank participants.* Lancet Public Health, 2018. **3**(7): p. e323-e332. <https://doi.org/10.1016/s2468-2667(18)30091-4>

8. Zhang, M., *Manual of psychiatric rating scale.* Hunan Science and Technology Press: Changsha, China, 1998: p. 213-227.

9. Zhang, S., Q. Qiu, S. Qian, et al., *Determining Appropriate Screening Tools and Cutoffs for Cognitive Impairment in the Chinese Elderly.* Front Psychiatry, 2021. **12**: p. 773281. <https://doi.org/10.3389/fpsyt.2021.773281>
